# IoT On-Board System for Driving Style Assessment

**DOI:** 10.3390/s18041233

**Published:** 2018-04-17

**Authors:** Bartosz Jachimczyk, Damian Dziak, Jacek Czapla, Pawel Damps, Wlodek J. Kulesza

**Affiliations:** 1 BetterSolutions S.A. Al. Grunwaldzka 103, 80-244 Gdansk, Poland; 2 3DPLab Do Studzienki 28, 80-227 Gdansk, Poland; dziak@3dplab.pl; 3 Blekinge Institute of Technology, Department of Applied Signal Processing, 371 79 Karlskrona, Sweden; jacaczap@gmail.com (J.C.); damps.p@gmail.com (P.D.); wlodek.kulesza@bth.se (W.J.K.)

**Keywords:** driver’s behavior, driving style, skills assessment, eco driving, embedded system, internet of things, real-time vehicle tracking

## Abstract

The assessment of skills is essential and desirable in areas such as medicine, security, and other professions where mental, physical, and manual skills are crucial. However, often such assessments are performed by people called “experts” who may be subjective and are able to consider a limited number of factors and indicators. This article addresses the problem of the objective assessment of driving style independent of circumstances. The proposed objective assessment of driving style is based on eight indicators, which are associated with the vehicle’s speed, acceleration, jerk, engine rotational speed and driving time. These indicators are used to estimate three driving style criteria: *safety*, *economy*, and *comfort*. The presented solution is based on the embedded system designed according to the Internet of Things concept. The useful data are acquired from the car diagnostic port—OBD-II—and from an additional accelerometer sensor and GPS module. The proposed driving skills assessment method has been implemented and experimentally validated on a group of drivers. The obtained results prove the system’s ability to quantitatively distinguish different driving styles. The system was verified on long-route tests for analysis and could then improve the driver’s behavior behind the wheel. Moreover, the spider diagram approach that was used established a convenient visualization platform for multidimensional comparison of the result and comprehensive assessment in an intelligible manner.

## 1. Introduction

Modern technologies pave the way to the objective assessment of professional skills. The simulation and real-time systems supporting such assessment can be found in evaluation of medical and security staff as well as many other professions where mental, physical, and manual skills are crucial. Each such solution focuses on specific measures for the assessed trade. For example, transportation companies search for a solution allowing objective driver skill assessment based on measures of the transportation cost including fuel consumption and fleet maintenance, driving safety, and delivery time. However, these factors are ambiguous and may be affected by external circumstances such as road conditions, traffic accidents and even a vehicle’s technical condition, which are independent of the driver. 

The problem can be solved by assessing the drivers based on their driving style, independent of external conditions. However, emerging technologies and methods, especially in remote sensing and Internet of Things (IoT), obtain information about driving parameters as well as external conditions to evaluate the person’s driving style. Such assessment can be used to grant professional drivers, who preserve passenger’s convenience or a cargo’s safety and consider economy along with eco-driving tips. Moreover, the insurance and car rental companies, with monitoring possibilities of the person’s driving style, could grant special discounts for those driving economically and safely. With the proper assessment program, driving schools could get information on trainees, i.e., ready for examination or more practice needed. Also, people who would like to improve their skills may gain from such a system showing their weaknesses to guide progress [1].

To address the problem of the objective assessment of driving style, we propose the vehicle monitoring system based on the IoT approach, using the car’s diagnostic port—OBD-II—and the designed and developed additional acquisition module using an accelerometer sensor and GPS module. Moreover, we propose sets of indicators and criteria, which can be used to comprehensively assess driving style. The indicators, which are associated with the vehicle’s speed, acceleration, jerk, engine rotational speed, and driving time, are used to assess three criteria of driving style: *safety*, *economy*, and *comfort*. The gathered multidimensional data are normalized based on expert results and then visualized and assessed using the spider diagrams approach. 

The modelled driving skills assessment method has been designed and implemented on a real car. For this purpose, an embedded On-board Measurement and Communication Unit (OMCU) has been developed. The system was then evaluated and verified experimentally. The obtained results prove the system’s ability to distinguish different driving styles, which are based on three assumed criteria consisting of eight indicators and categorized as ordinary, calm, aggressive or unnatural. Moreover, the spider diagram approach established the convenient visualization platform for multidimensional data, and the comprising comprehensive assessment is presented in an understandable manner. 

## 2. Survey of Related Works

The progress in technology enables users to assess the competence of professionals, such as medical staff or emergency serviceman. A methodological approach for tracking human movement and behavior resulted in a proposed system for firefighter’s skills and competence assessment [2]. The spider chart is chosen to visualize and graphically compare the results of the trainees with the expert. The result is quantified by the surface area of the polygon in the chart. 

The major interests in analysis of driving style using different systems and algorithms are comprehensively reviewed in [1], and they are not iterated here. The following review focuses on areas, which are directly related to the proposed solution: human and quality aspects of driving; driving style indicators and instrumentation, along with classification methods, however to avoid redundancy, in some references these areas cannot be separated.

### 2.1. Human, Social and Quality Aspects of Driving Styles 

A driving style can be described as a combination of driving abilities and behind-the-wheel behavior. Driving abilities depend on the driver’s knowledge, skills, and experience. The driver’s behavior reflects driving abilities, developmental factors, personality, demographic factors, biological features, perceived environment and driving environment [3,4,5]. All those factors influence driving tendencies: speeding, unsafe passing, impaired driving, and tailgating [3]. Studies show that young drivers have a higher rate of crashes and offences in comparison to experienced ones [4]. In a case of teen drivers, the presence of passengers inside the vehicle has an influence on crash risk, because of the distracting effect of social interaction during driving [5].

Psychologists identify seven different driving personalities depending on an interaction with other motorists and the driver’s own behavior [6]. There is even a claim the way that a driver holds a steering wheel reflects his personality [7].

As stated in the introduction, driving styles might have a great influence on transportation quality aspects such as safety, comfort, and economy. According to [8,9], safety on the road is of highest national importance and many road accidents might be prevented. Apart from using mobile devices and drunk-driving, a driver must also not exceed the speed limits. A driver should take into consideration the weather and road conditions and adjust the driving speed cautiously. Harsh accelerating and braking along with sharp cornering are recognized as dangerous behaviors. Driver’s fatigue is often ranked as a major factor of serious road crashes. The safety aspect of driving style is mentioned in almost all related publications. 

Economy is widely researched topic related to drivers’ assessment. Authors of [10] directly address their application to improving driving economy. In [11,12], authors proposed an eco-driving assistant to assesses the driver’s driving style considering both environmental and vehicle’s variables. Authors of [13] proposed to evaluate the long-term impact of an eco-driving training course. 

Apart from the economic aspects, a driving style has a big impact on comfort of the passengers traveling by cars, buses, or trams [1]. According to [14], the driver’s behaviors that may affect the passenger comfort experience are: uneven driving, heavy braking, sharp accelerating and harsh cornering. The author of [15] has proposed to model the driving style using the acceleration of the bus in three axes. The passengers were asked to grade their experience during the journey and meanwhile the data from the accelerometer were collected. The correlation between the acceleration in three axes and the passengers’ comfort experience was found.

### 2.2. Driving Style Indicators and Instrumentation

Several of Information and Communication Technology (ICT) solutions for increasing the safety and the economy of driving have been developed worldwide. An eco-driving assistant for Android is proposed in [11,12]. The solution is based on the use of a mobile phone, Bluetooth and OBD-II diagnostic port module. The system assesses the driver’s driving style considering both environmental and vehicle’s variables. To evaluate the relationship between fuel consumption and driving style, an expert system with Random Forest classification is run on the mobile device. In [16], simulations are conducted to analyze an impact of eco-driving behavior. It concludes that in normal and free traffic conditions, the eco-driving reduces CO_2_ emissions by 10–15%.

In [13], an on-board logging device is proposed to evaluate the long-term impact of an eco-driving training course. The collected data include the mileage, engine rotational speed, position of the acceleration pedal, gear selection and instantaneous fuel consumption. Ten participants have undertaken the four-hour course during the examination period. The project showed that based on the eco-driving tips, the mean value of fuel consumption for all drivers decreased by 5.8% and changes in the driving behavior were observed. 

Driving styles define a dynamic behavior of a driver on the road [17]. In this paper, the authors classify the driver’s style using the measure of how fast he accelerates and decelerates. The experiments were conducted using a vehicle simulation program. Applying the same normalized parameters, the driver is classified into one of three classes: calm, ordinary or aggressive.

There are available built-in-systems that assist drivers in eco driving, e.g., Scania Driver Support [18], which was developed for Scania trucks. The system analyses the data from the truck’s sensors and provides safety and eco driving tips to the driver.

There is a high potential in using the inertial sensors to differentiate between different drivers using features associated with acceleration, cornering, and braking behaviors. The authors of [19] propose Support Vector Machine and k-mean clustering as their training algorithms. In [20], authors applied k-nearest neighbors classification algorithm classifying driving style as aggressive or ordinary. 

In [21], several measures of driving style and their correlation with the predictability of the driver in different conditions are proposed. Drivers are distinguished as aggressive and non-aggressive based on the lateral and longitudinal accelerations and their derivatives with respect to time, called respective jerks. 

Different driving styles such as aggressive, calm and careful, and the division between them are discussed in [22,23]. The authors defined specific features, their indicators, and the possibility of measuring them.

### 2.3. Driving Style Classification Methods

In [24], a self-developed GPS-based device is used to collect driving parameters and send them to a server where Hierarchical Cluster Analysis (HCA) and Principal Component Analysis (PCA) are used to identify drivers’ behavior. This method may be valuable especially for those involved in fleet management, and it can be used to increase traffic safety. 

Using frequently occurring patterns of vehicle driving, such as a left or right turn or curve, stop or making a U-turn, the author presents a method for a driving event recognition using Hidden Markov Models (HMMs) [25]. The used data acquisition system collects information about the speed along with lateral and longitudinal accelerations from a vehicle. The research shows that HMMs are accurate and reliable for driving events recognition. Deng et al. proposed driving style classification using braking characteristics based on HMM [26].

Dynamic Time Warping (DTW) algorithm and a smartphone sensor fusion can be used in [27]. Neural Networks can be utilized to characterize the road type and the driving style [28].

Applications of some common classification methods for driving style assessment, such as Fuzzy Logic, Clustering, Bayesian approach, Decision Tree and others are presented in [1].

## 3. Problem Statement

From the review of related works, one can observe a rising interest on monitoring and assessing of motorists’ driving style, which defines driving skills and behind-wheel behavior. The advance in car embedded systems and an availability of standardized on-board diagnostic systems, provide data from the vehicle’s internal sensors, which indicate a driving style. The related works show different ways of assessing safety [8,9], economy [10,11,12,13], or comfort [14,15] using speed, speed limit, engine rotational speed, driving without rest and jerks. Often, the OBD-II diagnostic port, GPS module or mobile phone are used [11,12,13,15,18,24,27]. Nevertheless, none of the works combines all criteria into one system. Moreover, most of the assessments were done on a predefined route or by simulations. Furthermore, none of the solutions applies information about an actual speed limit of road. Additionally, usage of graphical visualization combined with driver’s assessment has not been reported. Although recognition and classification of the driver’s style is done in various manners however, there is no solution of quantitative evaluation of criteria and overall assessment as a single score.

The objective of this research is an objective driving style assessment method based on indicators, which are acquired from a measurement system monitoring the car’s dynamic driving parameters.

We assume that driving style assessment can be grounded on the three driving quality criteria: *safety*, *economy* and *comfort* [1]. These quality criteria can be estimated based on vehicle’s speed, accelerations and jerks in three axes, engine RPM and driving time. The applied indicators are normalized to the expert-based pattern as defined in Table 1.

The proposed embedded system is designed in accordance with the IoT concept where data acquisition is done using the available diagnostic port—OBD-II, along with an additional accelerometer sensor and GPS module. The Raspberry PI and GPRS modules provide data via the GSM network. Moreover, the spider diagram is assumed to be a suitable way to visualize and score the multidimensional assessment of driving style.

The main contribution of this work is a proposed method for driving skills assessment, considering more information than time of delivery and fuel consumption. Moreover, this work offers multi-quantitative visualization of the results using spider charts on a single diagram. Furthermore, the proposed solution is validated and verified based on real measurements and with real-world scenarios. The resulting system, due to its small size, low cost, simple architecture, and applicability to wide range of vehicles, can be easy customized to different commercial applications.

## 4. Driving Style Assessment Indicators and Criteria

The basic design stage of a driver style assessment system is to choose driving style criteria and corresponding measures that reflect interactions between the driver and a car along with the surrounding environment. In this approach, the driving style defines the driver’s skills and behavior behind the wheel. However, surrounding environmental aspects such as daylight condition, weather conditions in terms of precipitation and ambient temperature, road type and condition are not considered in this driver style assessment. However, they can be added to the assessment criteria easily and without special investment. 

Since the driving style assessment is to be used for different purposes and to make it more flexible, we propose the aforementioned assessment criteria: *safety*, *economy,* and *comfort*. Selection of these criteria is in line with references presented in Section 2 and with the results of the authors’ user-driven-design. The interviewed stakeholders have chosen these three criteria as most relevant for their applications.

These three criteria can be used exclusively to guide the drivers in improving their style and may be useful for risk analysis and professional skills assessment.

Each assessment criterion is quantified using various indicators, which are normalized. Some of them refer to established regulation standards, and others refer to expert’s ride and behavior measures. Selection of indicators is limited to the quantities accessible via the OBD-II socket of an ordinary personal vehicle along with the commonly used 3D acceleration and data from a GPS module. The applied eight indicators were defined based on reports presented in the Review of Related works [3,8,9,10,12,13,14,15,16,17,22], but also on our pre-study and -test. All indicators are estimated from measures acquired within a single test ride time *t_str_*, when *N* samples of the defined sampling rates are collected.

The criteria, corresponding indicators, symbols, and the proposed definitional equations are summarized in Table 1.

### 4.1. Indicators

Each of the eight proposed indicators is used for single, two and even three criteria. This means that some aspects of the criteria overlap or even include each other. 

The most common for all criteria is the *de- and accelerating ratio*, *jr_x_* (1), which indicates a driver’s tendency for aggressive driving. The indicator is obtained by analyzing the car’s dynamics represented by mean jerks in *x* axis, where jerk is understood as the first derivative of the car’s acceleration *a_x_* in respect to time. To assess if a driver does not accelerate or decelerate too rapidly, his mean value is related to respective *mean expert’s jerk* $\bar{Ex}$. The acceleration can be measured using conventional accelerometers.

*Bumping ratio*, *jr_z_* (2), is a measure of driver’s tendency to pass over speedbumps or road holes with too much speed. The indicator estimated from the mean jerk in *z* axis relates to the *mean expert’s jerk* $\bar{Ez}$. 

*Cornering ratio*, *jr_y_* (3), identifies if corners are taken smoothly and calm or sharply and fast, which indicates both driver’s skills and behavior. The indicator is measured as the mean jerk in *y* axis. To assess if the sharpness of the bend matches the speed, the mean value is related to *mean expert’s jerk* $\bar{Ey}$. 

*Driving time without rest ratio*, *dtr* (4), assesses if the driver follows the rule of resting after a recommended driving time. It can be mapped from engine uptime information, which shows time of driving without stopping and is available using OBD-II interface. This indicator is reasonable for a ride, which takes more than two hours.

*Car speeding ratio*, *spr* (5), is the average driving speed over the limit during the test ride. This value is calculated based on *M* samples of *current speed*, *CS*, of the *speed above the limit*, *SL*. However, to comprehensively assess the driver’s style, the speeding distribution is more informative. Therefore, apart from the mean value, the normalized standard deviation σ*_spr_* is also used.

*Car speeding duration ratio*, *spdr* (6), shows length of time the driver exceeds the speed limits. It is estimated by relating the number of samples *M* of the exceeding speed limit to all *N* samples of the test ride.

*Excessive engine rotational speed ratio*, *rsr* (7), assesses mostly an economical aspect of driving style. This mean value of revving is calculated based on *L* samples of *engine rotational speed*, *RERS*, exceeding the recommended value related to the recommended value *EERS*. However, to comprehensively assess this indicator, the excessive engine rotational speed distribution is needed. Therefore, apart from the mean value, the normalized standard deviation σ*_rsr_* is also used. These measures are acquired via diagnostic ports.

*Excessive engine rotational speed duration ratio*, *rsdr* (8), measures length of time the engine rotational speed was exceeded in comparison to the test drive time. Since short-term engine rotational speed overrun may sometimes be necessary, the time ratio of exceeded engine rotational speed is proposed.

### 4.2. Criteria 

The *driving safety criterion*, *SAFC*, is based on six indicators (1)–(6) [3,8,9,22]. Most of the ratios: bumping, *jr_z_*, de- and accelerating, *jr_x_*, along with cornering ratio, *jr_y_*, together with car speeding, *spr*, and car speeding duration, *spdr*, are measures of driving dynamics and may show a hazardous loss of tire friction. The first three indicators show how the driver’s behavior differs from the expert’s one, which is a pattern of safe driving. Two other indicators, i.e., the car speeding and car speeding duration are used to estimate how much and how frequently, the driver exceeds the speed limits, which are laid down by regulations.

Apart from behavioral factors, a habit of resting-less driving time is important for safety. Therefore, driving time without rest ratio, *dtr*, is included in the *safety* criterion.

*The economy criterion*, *ECOC*, is relevant not only from an economy point of view but also due to the sustainability issue. The criterion is very substantial for the professional drivers and their employers. The *ECOC* can be based on the car’s engine rotational speed and how much it exceeds the eco-driving standards, which are directly related to fuel consumption [10,11,12].

Bumping ratio, *jr_z_*, indicates a habit of fast passing over the road speedbump or holes, which lead to damages and shortens life time of the car’s parts. Furthermore, an excessive usage of brakes and throttle, indicated by de- and accelerating ratio, *jr_x_*, affects both fuel combustion and wear of parts, i.e., tires and brake pads [12,13,17].

Long driving with high engine rotational speed, measured *by excessive engine rotational speed* ratio, *rsr*, affects fuel consumption [29,30]. Moreover, when the driver exceeds the recommended engine rotational speed for longer periods, it indicates uneconomical driving on too low of a gear ratio [13]. 

*The comfort criterion*, *COMC*, measures passenger’s convenience and the cargo’s safety in terms of its displacement and damage. The criterion is assessed using four indicators of driving smoothness, which mostly depends on ways of deceleration and acceleration, i.e., turning too fast or hesitantly [14,15]. Moreover, the manner of driving through speedbumps or potholes is important for the passenger’s experience. All these events can be indicated by acceleration measures in the car’s three axes and jerk calculations given by (1)–(3).

Apart from the safety issue, the long drive without a rest can be inconvenient, both for the driver and passengers. Therefore, the *driving time without rest ratio* indicator, *dtr* (4), contributes to the *comfort* criterion.

### 4.3. Assessment

**Figure 1:**
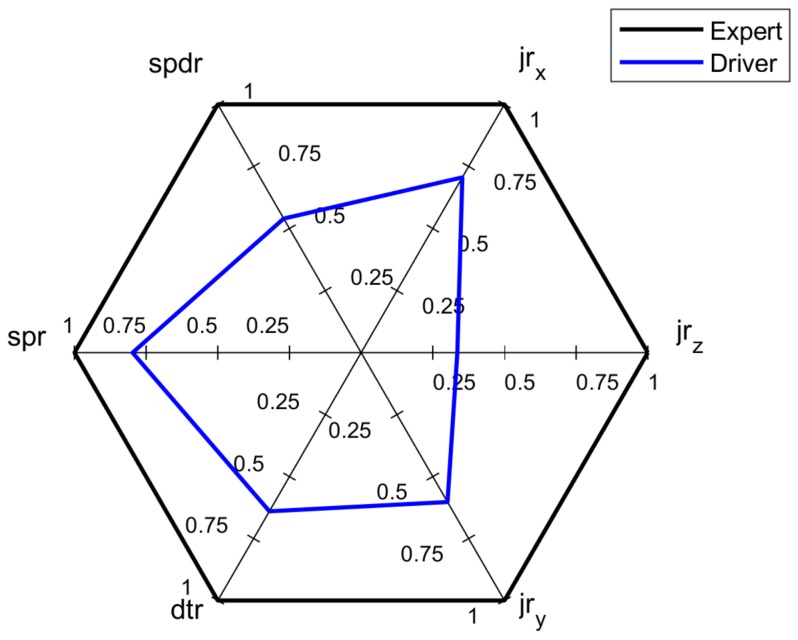
Example of spider diagram of *SAFC.*

To objectively assess and visualize a multidimensional problem of driving style assessment, the spider diagram approach [2] is proposed. This driving style assessment is based on analysis of *SAFC*, *ECOC* and *COMC* criteria, with an overall assessment including each indicator. Figure 1 shows an exemplary assessment based on *SAFC*, with six indicators *jr_x_*, *jr_z_*, *jr_y_*, *dtr*, *spr* and *spdr*, where all of them have the same weights. The black contour line represents the expert’s scores of each indicator and the blue contour line depicts assessed driver’s results. 

Analyzing the surface area of the resulting blue hexagon, it is possible to depict the driver’s *safety criterion score* $DSC_{s}$ that can be calculated as a sum of six triangles from the formula:

(9) $$DSC_{s}=\frac{1}{2}\times \sin\left(60{}^{\circ}\right)\times \left(jr_{x}\times jr_{z}+jr_{z}\times jr_{y}+jr_{y}\times dtr+dtr\times spr+spr\times spdr+spdr\times jr_{x}\right)$$

The driver’s $DSC_{s}$ related to an expert safety criterion score, $ESC_{s}$ forms the normalized safety criterion score, $NSC_{s}$:

(10) $$NSC_{s}=\frac{DSC_{s}}{ESC_{s}}=\left(jr_{x}\times jr_{z}+jr_{z}\times jr_{y}+jr_{y}\times dtr+dtr\times spr+spr\times spdr+spdr\times jr_{x}\right)/6$$

A similar assessment method may be used for driver’s *normalized economy criterion* and *comfort criterion scores*, $DEC_{s},$ $DCC_{s},$ respectively:

(11) $$NEC_{s}=\frac{DEC_{s}}{EEC_{s}}=\left(jr_{x}\times jr_{z}+jr_{z}\times rsr+rsr\times rsdr+rsdr\times jr_{x}\right)/4$$

(12) $$NCC_{s}=\frac{DCC_{s}}{ECC_{s}}=\left(jr_{x}\times jr_{z}+jr_{z}\times jr_{y}+jr_{y}\times dtr+dtr\times jr_{x}\right)/4$$

where $EEC_{s}$ and $ECC_{s}$ are the expert’s scores for *safety* and *comfort* criteria respectively.

The proposed overall assessment score composed of all eight indicators, and the driver overall normalized final score $NO_{s}$ can be calculated as:

(13) $$NO_{s}=\frac{DO_{s}}{EO_{s}}=\left(jr_{x}\times jr_{z}+jr_{z}\times jr_{y}+jr_{y}\times dtr+dtr\times spr+spr\times spdr+spdr\times rsr+rsr\times rsdr+rsdr\times jr_{x}\right)/8$$

where $DO_{s}\;\mathrm{and}\;EO_{s}$ are driver’s and expert’s overall score, respectively.

The proposed graphical visualization facilitates a comparison of the latest results of assessment of driving style with the previous ones, showing which indicators of driving style have been enhanced and which still need to be improved.

## 5. System Architecture

**Figure 2:**
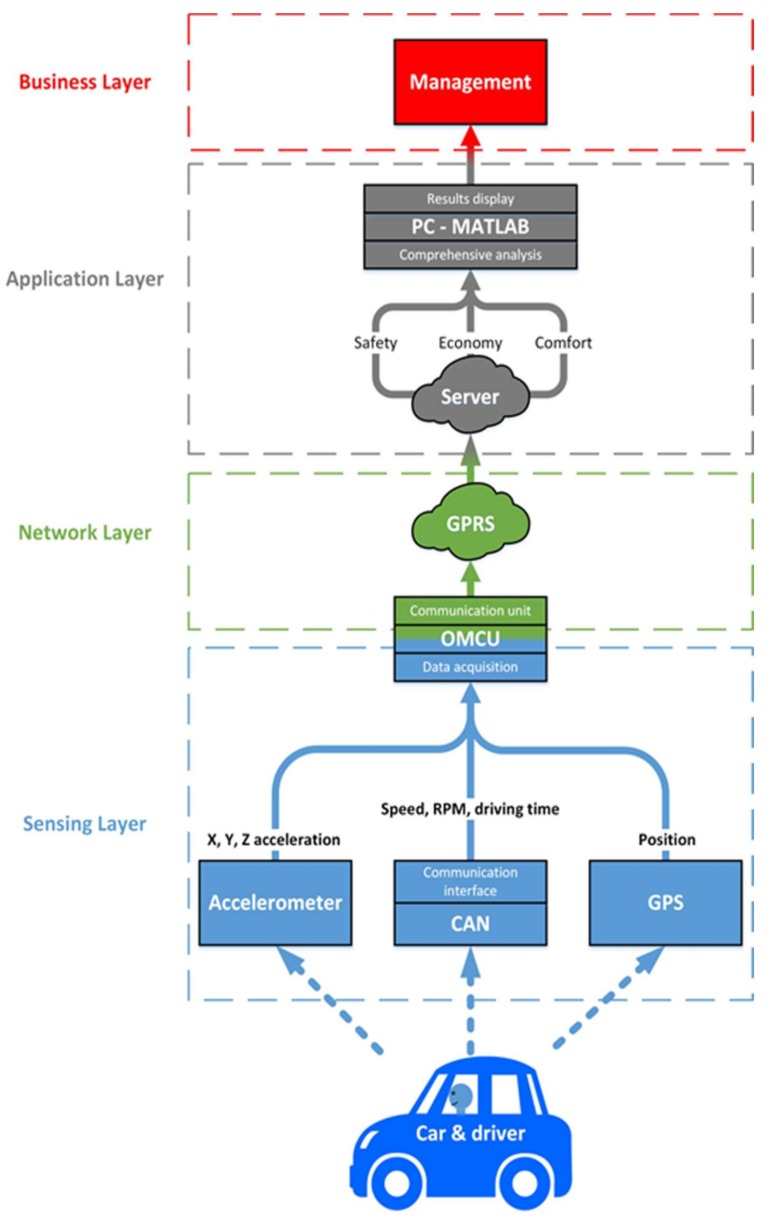
Architecture of the IoT-based driving style assessment system.

The driving style assessment system is designed according to the IoT reference model [31] and consists of the four functional layers: *Sensing*-, *Network*-, *Application*-, and *Business-layer*. The functionalities of each layer are supported by the relevant hardware and software. The system structure is presented on the block diagram in Figure 2.

The *Sensing-layer* is responsible for data collection during the driving, data pre-processing including filtering and edge computing. The functionalities are executed at OMCU hardware.

A part of OMCU hardware performs communication functionalities, therefore, it also belongs to the *Network-layer*. The included communication module provides data for further analysis via a mobile network through GPRS. All standardized communication protocols of data transmission belong to the Network-layer of the model.

In the *Application-layer*, high-level data processing such as filtering, data synchronization and calculations of the driving indicators are performed. In this layer, the cloud-level comprehensive analysis and results visualization are realized. These processes are supported by dedicated algorithms from MATLAB.

The final *Business-layer* includes the management and decision-making functions, where relevant algorithms are implemented. At this layer, the previously acquired and pre-processed data are used to deliver information required by specific applications. The applications can also aim for a specific business purpose, such as risk analysis or assessment of driving style required by a car rental or insurance company.

## 6. System Implementation

Hardware and software implementations have been executed within the project frame and are described in detail in the following sections.

### 6.1. Hardware Implementation

The On-board Measurement and Communication Unit, OMCU, is the main hardware system, which acquires data and communicates with the server, as shown in Figure 2. It consists of a single-board computer Raspberry Pi and expansion modules: the accelerometer, GPS module, GPRS module and real-time clock. Additionally, the OMCU is integrated with the OBD-II adapter via a Bluetooth module of the Raspberry Pi.

The single-board computer Raspberry Pi 3 constitutes the main component of the OMCU embedded device. It runs on a Debian-based Linux operating system called Raspbian. On the Raspberry Pi, data are acquired, stored, and processed. It can be powered either from the car’s 12 V socket via USB adapter or a power bank.

For the acceleration measurement, the 3-axis ADXL345 accelerometer with selectable measurement ranges is applied. For this application, ±4 g range is chosen, and the data rate can be between 0.1 Hz and 3200 Hz. Based on a conducted empirical test, the data rate of 12.5 Hz is found as sufficient for driving style analysis. It measures both dynamic accelerations resulting from motion or shock and static accelerations, including gravity. The module is connected to Raspberry Pi using I2C bus. 

The actual car’s position is determined via the NEO-7M-C GPS receiver module, which communicates with Raspberry Pi by a Universal Asynchronous Receiver and Transmitter (UART) interface applying the NMEA 0183 standard.

To acquire in real-time the vehicle’s variables such as speed, engine load and engine rotational speed, the iCar III OBD Scan-tool adapter is applied. This module also enables reading, erasing, and displaying of the diagnostic codes, which are used in troubleshooting. The adapter is plugged into the vehicle’s OBD-II socket and communicates with Raspberry Pi via the Bluetooth module.

None of the available Raspberry Pi models consists of a built-in real-time clock, so they are unable to keep track of daytime without an Internet connection. Therefore, a real-time hardware clock DS3231 with a battery backup is added to the system using the I2C bus to provide the current time offline.

The GSM/GPRS dual SIM-c-uGSM μ-shield v.1.13 module is used to enable a communication between the computer and Internet. The GPRS module provides a low speed high range mobile Internet connection. However, it requires a SIM card and an additional 250 mAh LiPo battery due to possible current spikes higher than 1.5 A. The module is connected to the Raspberry Pi using the serial communication protocol via USB interface.

### 6.2. Software Implementation

According to the IoT architecture presented in Figure 2, the software part of the system is implemented at three IoT layers, i.e., Sensing-, Network- and Application-layer. For Sensing- and Network-layers, the OMCU’s application program has been developed in Python programming language and runs on Raspberry Pi in the auto start mode. The program is divided into threads working independently and simultaneously in an infinite loop. Each thread is responsible for different functions of the system: collecting and sending data to the server and control of the whole system. In the IoT Application-layer, the collected data are sent to the server via OMCU and processed in MATLAB R2017a environment.

**Figure 3:**
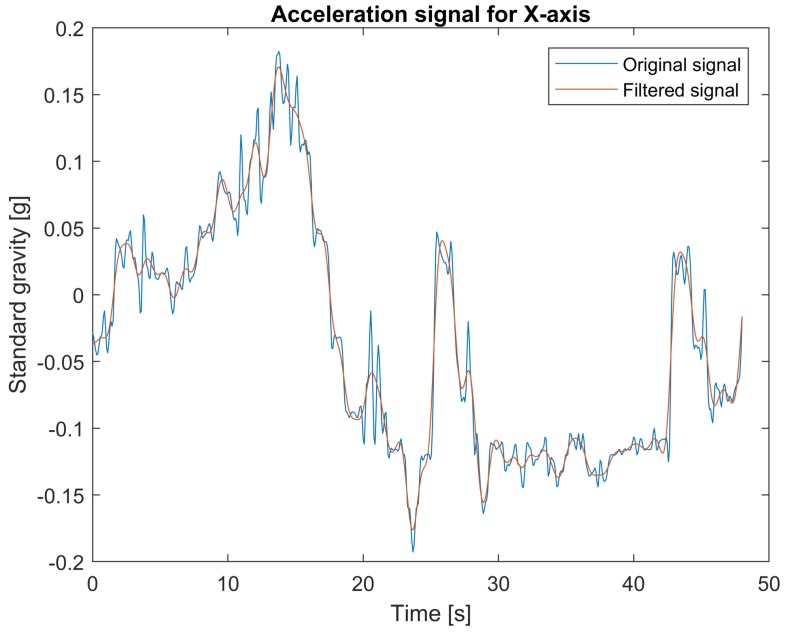
Original and filtered acceleration signal for *x*-axis.

**Figure 4:**
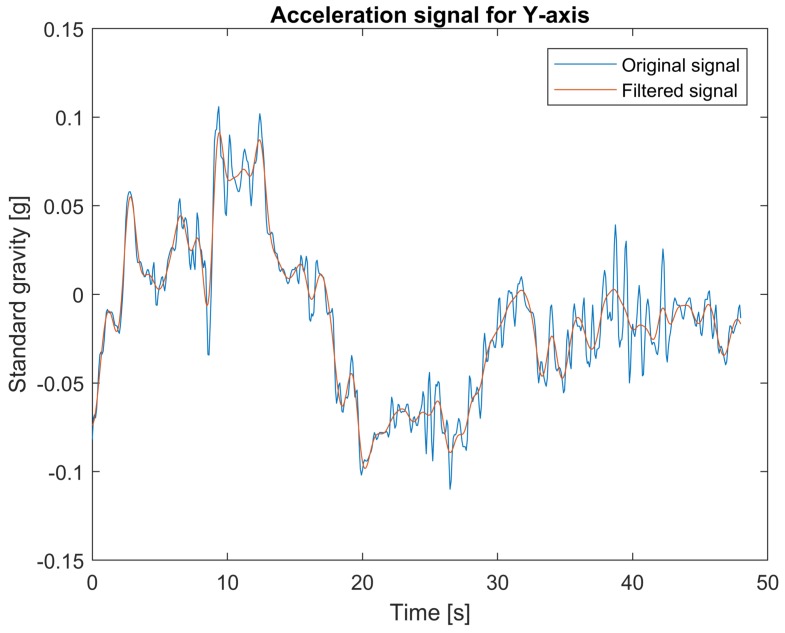
Original and filtered acceleration signal for *y*-axis.

**Figure 5:**
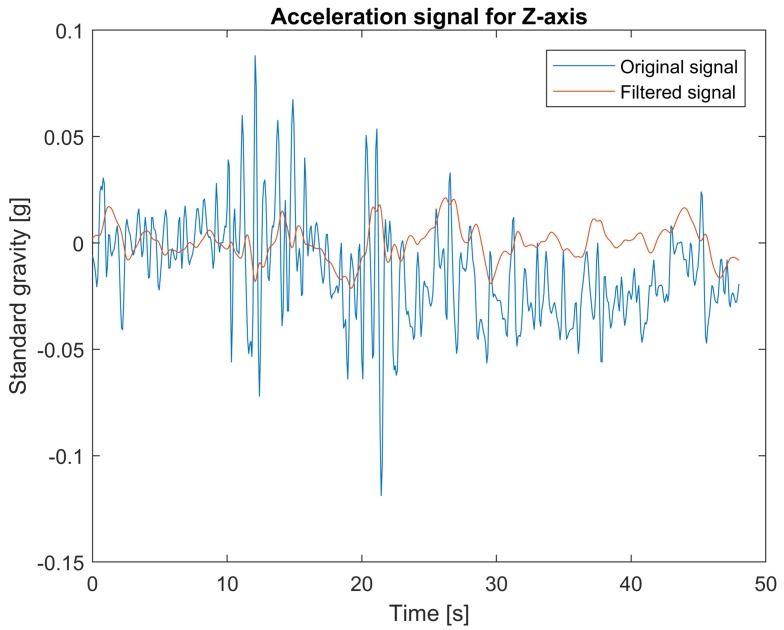
Original and filtered acceleration signal for *z*-axis.

The data from the accelerometer are filtered using two types of filters: low-pass filters for measurements in *x* and *y* axes and band-pass filter for measurements in *z* axis. The 4th order lowpass Butterworth filter with cut-off frequency at 0.8 Hz is applied. For the case of the *z* axis, useful information needs to be extracted from the signal corrupted by the vehicle vibration response component varying between 1 Hz and 2 Hz [32]. Another disturbance component of low frequency is caused by landform. Therefore, to clear out these components, a 4th order bandpass Butterworth filter with cut-off frequencies 0.1 Hz and 0.8 Hz is applied. The filters’ parameters were chosen empirically. Figure 3, Figure 4 and Figure 5 show examples of accelerometer measurements for *x*, *y*, and *z*-axes respectively, before and after filtering.

Based on the vehicle’s GPS position, the road speed limits (needed to estimate an exceeded speed limit), are received from a dedicated API with OpenStreetMap.

### 6.3. Embeded System Prototype

**Figure 6:**
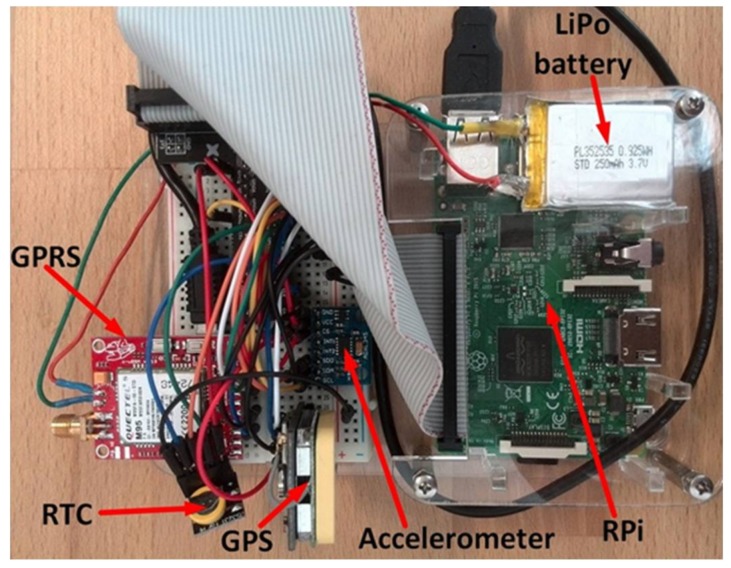
The OMCU prototype.

To interconnect all system parts, the solderless breadboard with 400 connection points is used. The GPIO breakout expansion board is connected to the Raspberry Pi by a ribbon cable. All expansion modules are jointed to the appropriate pins using jump wires. The breadboard is mounted on the Raspberry Pi plastic case. The photo of the assembled device is presented in Figure 6. 

**Figure 7:**
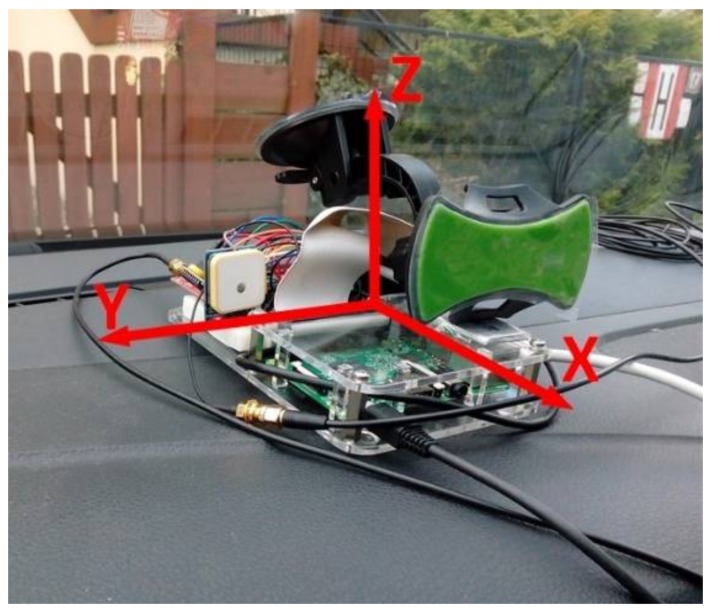
Device prototype mounted in the car, with depicted accelerometer axes.

The developed OMCU device was mounted on the test car’s dashboard as shown in Figure 7. The accelerometer measurements are sensitive to the device location, i.e., matching between the device’s axis and the car’s axis significantly affects the measurements. Therefore, mounting of the device for each test was very accurately done. The final system version consists of an automated start-up axes calibration regardless of the device’s placement.

## 7. Evaluation and Verification

The developed embedded system was evaluated by tests of multiple drivers on the same short route of 16 km. All defined indicators were measured to assess the criteria of *safety*, *economy*, and *comfort*, as well as an overall assessment. Then, the system was verified on the long-route test of 325 km, when the same parameters were assessed.

The evaluation and verification tests were conducted using the same car, Opel Astra H 2005, 1.6, 16 V, 105 HP equipped with a manual gearbox. During the test drives, the basic indicators of speed, engine rotational speed and engine uptime were obtained through an OBD-II interface, while the indicators of accelerations in *x*-, *y*- and *z*-axes and car’s location were collected by the acceleration and GPS expansion modules, respectively.

**Figure 8:**
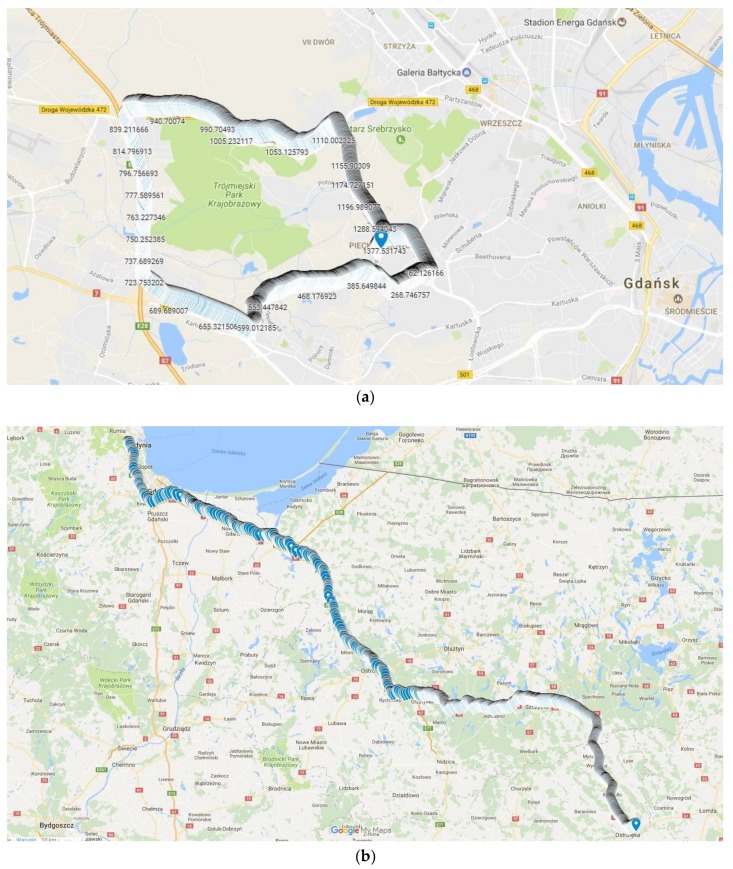
The GPS tracker of test route of (**a**) evaluation; (**b**) verification.

The evaluation was done by assessing the driving style of five drivers who imitated different driving styles described in Table 2. The test data set is limited because our validation is based on a case study approach, since the proposed assessment method is based on expert approach and does not apply any statistical model. Moreover, the results prove usefulness of the proposed solution in a sense that the diversities in scores among different driving styles are very distinguishable. Due to safety reasons we could only imitate/simulate driving styles, since a drunk, drugged, or sick person is not allowed to take such test. This kind of limitations is common in behavioral sciences. To map the expert’s behavior, a professional driver took the same route. The short route test included different road types, i.e., highways, ramps, and urban streets. Figure 8a depicts an example route from the GPS tracker; the positions are labelled with the driving time in seconds. Depending on the driver, each test lasted from 21 min to 26 min.

**Figure 9:**
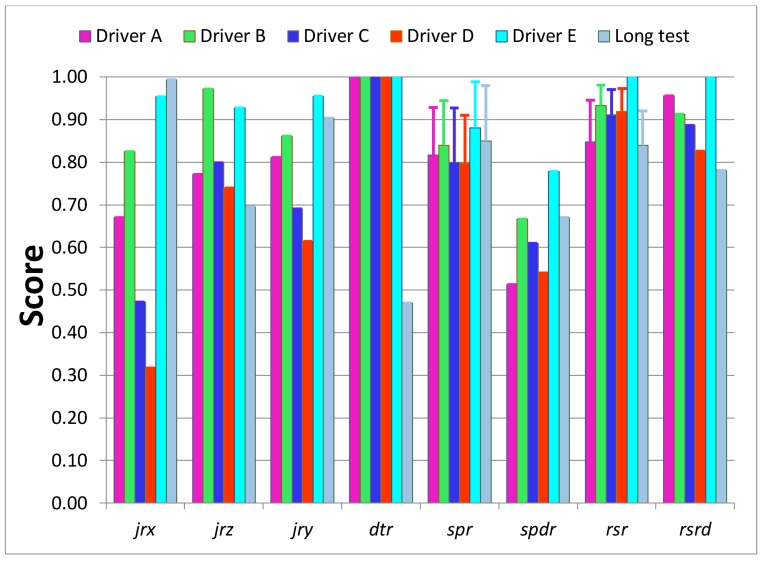
Bar graph of normalized indicators values from the test rides, where ‘1’ is the expert level.

The test data were used to calculate all indicators apart from the *dtr*, which is set to the maximum score one since the tests were shorter than two hours and rest was not required. The test results, summarized in Table 3 and on the bar-diagram in Figure 9, show that the used indicators clearly differentiate the driving styles. Drivers D and C, who simulated *unusual* and *aggressive* styles obtained the lowest values of all indicators, while the Driver E, who simulated *calm* driving style almost reached the *expert’s* level. 

The *jr_x_* is the most diversified of all indicators and has values of 0.95 and 0.32 for simulated *calm* and *unusual* driving styles, respectively. The least varied indicator is *spr* with values between 0.80 and 0.88 for Drivers D, C and E, respectively. It is noticeable that *std* of *rsr* for Driver A is much greater than others, which may indicate that he was less consistent with using the gas pedal. Also, *std* of *spr* for Driver C and Long-route test may indicate some inconsistency in driving speed.

**Figure 10:**
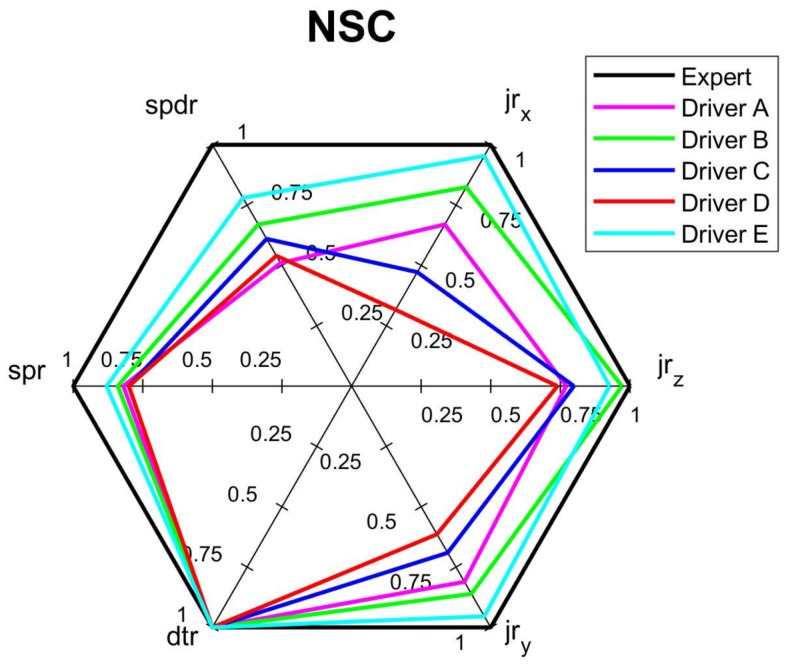
Spider diagram for *safety* criterion of short routes (*dtr* indicator is not distinct due to short time of the test).

**Figure 11:**
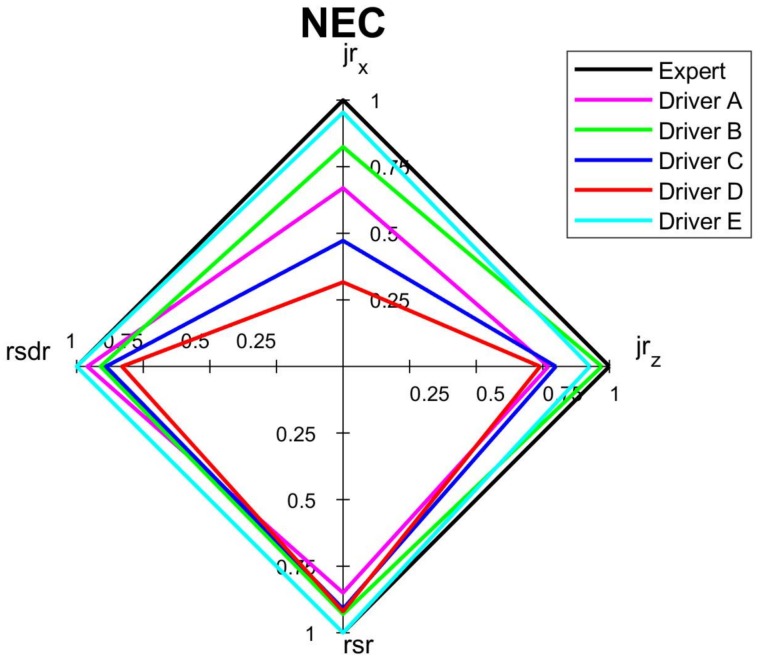
Spider diagram for *economy* criterion of short routes.

**Figure 12:**
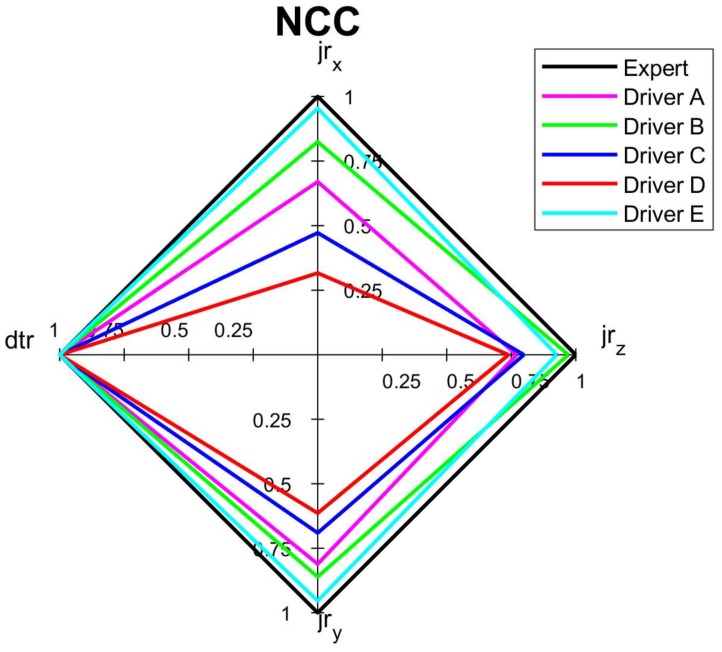
Spider diagram for *comfort* criterion of short routes (*dtr* indicator is not distinct due to short time of the test).

**Figure 13:**
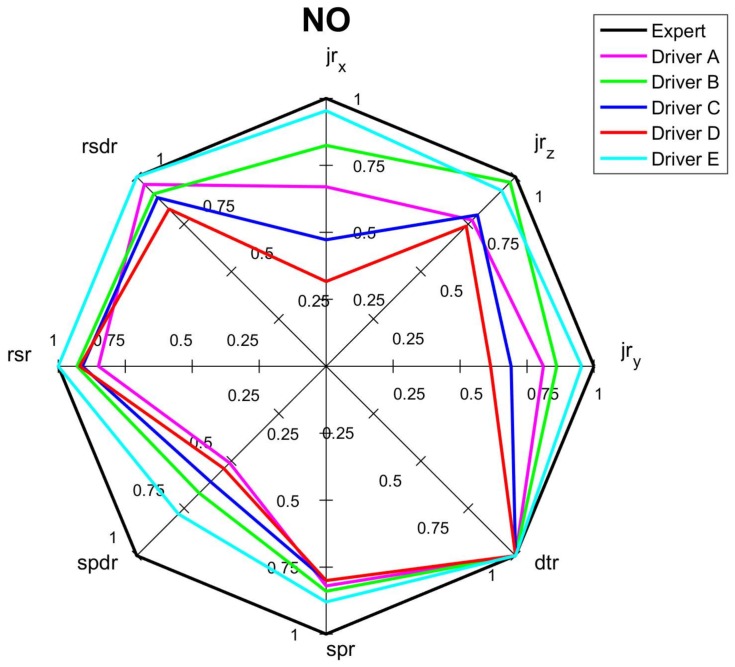
Spider diagram for overall scores (*dtr* indicator is not distinct due to short time of the test).

**Figure 14:**
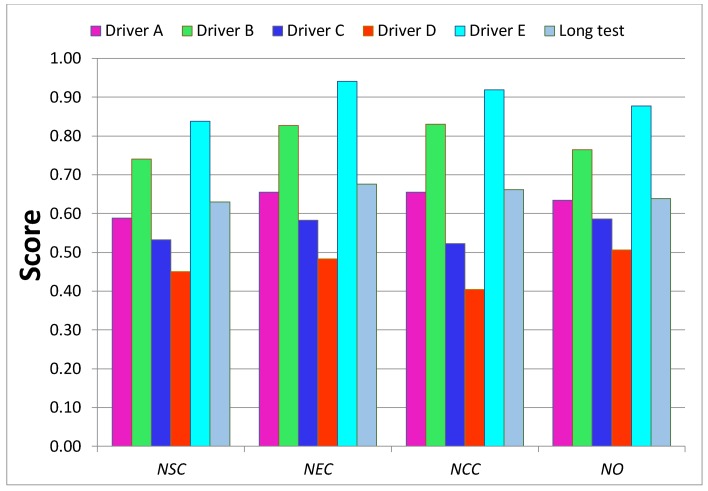
Bar graph of normalized criterion scores from the test rides, where ‘1’ is the expert level.

The comprehensive assessments of each criterion and overall assessment by the spider diagrams, where the relevant normalized indicators are mapped, are shown on Figure 10, Figure 11, Figure 12 and Figure 13. Moreover, Figure 14 summaries the normalized scores of each test driver for all criteria and the overall score.

The results of the normalized criterion scores show that Driver E, called *calm*, has the closest match to the *expert’s* driving style map. This is especially the case for *economy NEC_S_* and *comfort NCC_S_* criteria along with the overall score *NO_S_* of values 0.94, 0.92 and 0.88, respectively. The worst scores of all three criteria were of Driver *D*, who imitated the behavior of a sick or affected person and obtained only 0.45, 0.48 and 0.40 for *safety*, *economy,* and *comfort* criteria respectively, along with an overall score of 0.51. Driver *C*, following the *aggressive* style, significantly deviates from the *calm* and *expert* drivers. However, the *ordinary* driving of A does not have much better results than aggressive driving, especially for *safety* criterion and overall score, which can depict that his style was at limits of *ordinary* style. It is worth noticing that in most cases the *NOs* averages the other criteria, however in a case when just one indicator especially differs from the others, the effect of this indicator can be reduced in the overall score. It is a case of Drivers C and D whose *jr_x_* are relatively low compere to other drivers, nevertheless their effect on the final score is reduced.

The designed system was verified on the long-route test of 325 km, including typical types of roads. In Figure 8b, the road is mapped from a GPS tracker of four-hour trips. The results included in Table 3 and Figure 9 show that on the long-route test, with majority of highways and freeways, the changes of speed (driving dynamic) measured by the *jr_x_*, are less than in an urban environment. One of the lowest scores of 0.70 for the *dtr* indicator depicts the lack of rest since the assessed driver stops the car just once over a four-hour driving period. Another low score of *jr_z_* identifies that the driver tends to pass over speedbumps or road holes with high speed. The indicators *spr* and *spdr* of 0.85 and 0.67, respectively, show the driver slightly exceeded recommended speed limits but he has tendency to speed for a longer time. Also, the *rsdr* value of 0.78 is lower than corresponding values of the evaluation tests because driving on highways and freeways is characterized by high speed causing the excessive rotational speed.

**Figure 15:**
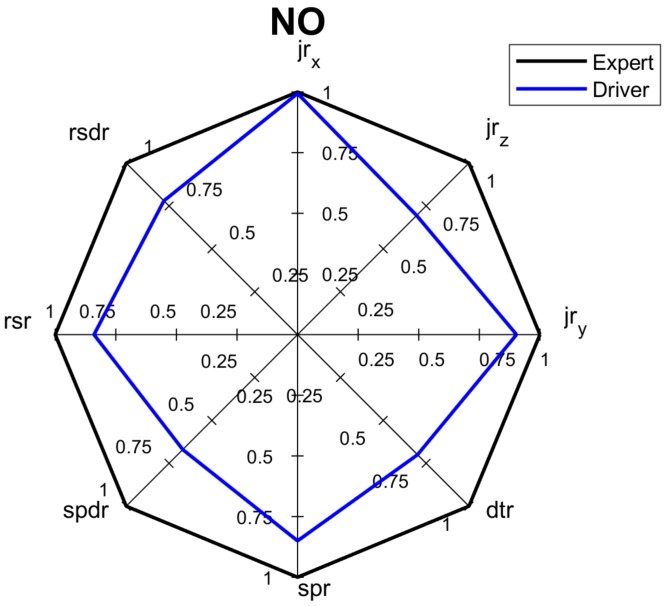
Spider diagram for overall score of long ride.

The low values of *dtr* and *jr_z_* measured during the long-route test greatly influenced the *NSC* and *NCC* of 0.63 and 0.66, respectively. Even *economy* criterion *NEC* of the long-route test show low value. The overall score of this test is also worse than one would expect. Figure 15 shows the spider diagram for the long-route test, which can be used by the driver to analyze his skills and learn how to improve his driving style. 

## 8. Discussion

To evaluate the proposed assessment method, test rides were conducted by five different drivers and an additional verification test drive have been performed. The experiments provide real measurement data and were carried out in real-world scenarios.

The validation results show that the best normalized overall score of driving style, 0.88, was obtained by the Driver E, which imitated *calm* driving style. The worst normalized overall score, 0.51, was obtained by the Driver D who drove unnaturally imitating the driving style of a sick person. Driver C who was driving aggressively scored 0.59. Drivers A and B were asked to drive naturally, however, their results are different since Driver A obtained results of 0.63, while Driver B scored 0.77. 

The biggest difference of the normalized overall score of 37% is between Driver E and Driver D, which shows how significantly the applied indicators vary among different driving styles. The differences between these two most distinctive drivers are even greater in terms of normalized safety, economy, and comfort scores of 39%, 46% and 52% respectively. Such gap in scores between the drivers proves that the analysis of driving style could be based on very distinguishable measures.

Comparing disparity of different normalized scores of driving quality criteria, one can see that the biggest diversity among tested driving styles appears for *comfort* criterion, 52%, while the *safety* criterion varies least of all (39%, from 45% to 84%). Furthermore, all considered driving quality criteria show a bigger diversity than the normalized overall scores.

Analysis of diversities among the applied indicators show that they vary from 63% to 8%. The *jr_x_* is the most diversified of all indicators with extreme values of 0.95 and 0.32 for simulated *calm* and *unusual* driving styles, respectively. The least varied indicator is *spr* with values between 0.80 achieved by Drivers C and D and 0.88 scored by Driver E. Therefore, the indicators can be ordered from the most to least sensitive as: *jr_x_*, *jr_y_*, *spdr*, *jr_z_*, *rsdr*, *rsr*, *spr.*

The proposed embedded system and assessment method were experimentally verified on the long-route test. The normalized overall score of this driving style was assessed for 0.64, which corresponds to *ordinary* driving style. Furthermore, all normalized criteria, i.e., *safety*, *economy* and *comfort* can be also considered in ordinary range. Moreover, the long-route test results show high similarity to the results of Driver A in all considering criteria. If the *dtr*, which indicates time of stops for a four-hour driving period, were better, the normalized overall score of driving style could reach 0.70.

Figure 10, Figure 11, Figure 12, Figure 13 and Figure 14 visualize the multi-variable quantities of driving style in a clear and legible way. Moreover, this approach facilitates a comparison of the driver’s score with an *expert* one, with other drivers and even with previous scores of the same driver. Therefore, among other applications, the designed system could be used by insurance or rental companies for awarding discounts to drivers of correct driving style. The proposed assessment approach can be customized to different needs and applications.

## 9. Conclusions

A system consisting of the embedded OMCU device for assessing driving style has been designed and developed according to the IoT concept and user-driven-design approach. The measurement data are acquired from the diagnostic port—OBD-II—additional accelerometer sensor and GPS module. 

The prototyped system used for driving style assessment was validated experimentally in real-world scenarios. 

The evaluation experiments conducted by five drivers on short routes proved that it is possible to differentiate and assess the person’s driving style in terms of *safety*, *economy,* and *comfort* criteria. The normalized scores of criteria are estimated based on the eight measurable indicators such as: normalized *de-* and *ac-celerating*, *bumping*, *cornering*, *driving without rest*, *car speeding*, *car speeding duration*, *excessive engine rotational speed* and *excessive engine rotational speed duration ratios*. 

The analytical assessment of driving style based on criterion and overall score visualization using a spider diagram approach can be easily customized by users according to their application need. 

The presented solution can be extended by implementing big-data statistical methods with a representative database of drivers and corresponding driving styles. Along with statistical analysis, an automatic data visualization engine can be applied. Such a module allows visualization of the driver’s assessment in the broader context. This feature could be especially desired by analysts and managers and may be used for business decisions. The proposed approach could be complemented by recognition of drivers’ dangerous behavior. 

The designed OMCU system could be supplemented with additional vision and noise sensors used to monitor the external driving conditions such as weather, road conditions, along with using more parameters from the car’s on-board computer e.g., acceleration pedal position or belt fastening. A customization and adjustment of the system prototype to other vehicles such as trains, trams, and busses would extend its application field.

## Figures and Tables

**Table 1:** The *safety*, *economy* and *comfort* criteria and corresponding indicators, their symbols, units, and definitions.

Criterion	Indicator	Symbol	Definition	
Comfort, Economy, Safety	De- and accelerating ratio	*jr_x_*	$\left(\frac{N\cdot \bar{Ex}}{\sum_{i=1}^{N}\left|\frac{\Delta a_{xi}}{\Delta t}\right|}\right)$	(1)
	Bumping ratio	*jr_z_*	$\left(\frac{N\cdot \bar{Ez}}{\sum_{i=1}^{N}\left|\frac{\Delta a_{zi}}{\Delta t}\right|}\right)$	(2)
Comfort, Safety	Cornering ratio	*jr_y_*	$\left(\frac{N\cdot \bar{Ey}}{\sum_{i=1}^{N}\left|\frac{\Delta a_{yi}}{\Delta t}\right|}\right)$	(3)
	Driving time without rest ratio	*dtr*	$\frac{2\cdot N_{os}}{t_{str}}$	(4)
Safety	Car speeding ratio	*sp*r	$\left(\frac{1}{M}\overset{M}{\underset{i}{\sum }}\frac{SL_{i}}{CS_{i}}\right)\pm \sigma_{spr}$	(5)
	Car speeding duration ratio	*spdr*	$\left(1-\frac{M}{N}\right)$	(6)
Economy	Excessive engine rotational speed ratio	*rsr*	$\left(\frac{1}{L}\overset{L}{\underset{p}{\sum }}\frac{ERSR}{EERS}\right)\pm \sigma_{rsr}$	(7)
	Excessive engine rotational speed duration ratio	*rsdr*	$\left(1-\frac{L}{N}\right)$	(8)

Where: *N*—a number of samples acquired within a single test ride; $\bar{Ex}$, $\bar{Ey}$, and $\bar{Ez}$—mean expert’s values of ratios of de- and accelerating, cornering and bumping ratios respectively; $\Delta a_{xi}$, $\Delta a_{yi}$ and $\Delta a_{zi}$—successive accelerations in *x*-, *y*- and *z*-axes respectively; Δt—sampling ratio; $N_{os}$—a number of rest stops during the test ride, $t_{str}$—time of single test ride; M—a number of samples of exceeding speed limits; SL and CS—speed limit and current speed respectively; L—a number of samples of exceeding recommended engine rotational speed; ERSR and EERS—recommended engine rotational speed and difference between engine rotational speed and recommended one respectively.

**Table 2:** Test rides and their descriptions [1,22].

Driver	Simulated Driver Style	Driving Style Description
A	Ordinary	- driving in a common, ordinary way; - medium dynamic of braking; - accelerating and deaccelerating regularly; - not too many speeding events; - turning smoothly.
B		
C	Aggressive	- driving fast and sharp braking; - accelerating and deaccelerating rapidly; - many speeding incidents; - maintaining high speed while turning.
D	Unusual	- simulating sickness or unnatural behavior; - braking unexpectedly; - accelerating and deaccelerating suddenly; - alternately low and high speed; - turning inconsistently and aggressively.
E	Calm	- driving calmly; - avoiding excessive braking; - accelerating and deaccelerating smoothly; - never exceed speed limits; - turning smoothly.

**Table 3:** Indicator values for the test rides.

Indicator	Driver A	Driver B	Driver C	Driver D	Driver E	Expert	Long-Route Test
*jr_x_*	0.67	0.82	0.47	0.32	0.95	1.00	0.99
*jr_z_*	0.77	0.97	0.80	0.74	0.93	1.00	0.69
*jr_y_*	0.81	0.86	0.69	0.61	0.95	1.00	0.90
*dtr*	1.00	1.00	1.00	1.00	1.00	1.00	0.70
*spr* (*std*)	0.82 (0.11)	0.84 (0.11)	0.80 (0.13)	0.80 (0.11)	0.88 (0.11)	1.00 (0.00)	0.85 (0.13)
*spdr*	0.51	0.66	0.61	0.54	0.77	1.00	0.67
*rsr* (*std*)	0.85 (0.10)	0.93 (0.05)	0.91 (0.06)	0.92 (0.05)	1.00 (0.00)	1.00 (0.00)	0.84 (0.08)
*rsdr*	0.95	0.91	0.88	0.82	1.00	1.00	0.78

## Abbreviations

The following abbreviations are used in this manuscript:

COMC	Comfort Criterion
DCC	Driver’s Comfort Criterion
DEC	Driver’s Economy Criterion
DO	Driver’s Overall
DSC	Driver Safety Criterion
DTW	Dynamic Time Warping
ECC	Expert Comfort Criterion
ECOC	Economy Criterion
EEC	Expert Economy Criterion
EO	Expert’s Overall
ESC	Expert Safety Criterion
HCA	Hierarchical Cluster Analysis
HMMs	Hidden Markov Models
ICT	Information and Communication Technologies
IoT	Internet of Things
NO	Normalized Overall
NCC	Normalized Comfort Criterion
NEC	Normalized Economy Criterion
NSC	Normalized Safety Criterion
OMCU	On-board Measurement and Communication Unit
PCA	Principal Component Analysis
RPM	Revolutions Per Minute
SAFC	Safety Criterion
UART	Universal Asynchronous Receiver and Transmitter
